# Falling Through the Cracks: The Need to Include Acute Pancreatitis in Risk Assessment Models for Acute Deep Venous Thrombosis

**DOI:** 10.7759/cureus.12056

**Published:** 2020-12-13

**Authors:** James Kamau, Elisabeth Paul, Mathai Chalunkal, Richard Snyder, Douglas S Corwin

**Affiliations:** 1 Internal Medicine, St. Luke's University Health Network, Easton, USA; 2 Internal Medicine, St. Luke’s University Health Network, Easton, USA; 3 Pulmonary and Critical Care, St. Luke’s University Health Network, Easton, USA

**Keywords:** pancreatitis, dvt, vte, risk assessment

## Abstract

Acute pancreatitis is an inflammatory condition caused by an insult to the pancreas. Pancreatitis is associated with local and systemic complications such as splenic vein thrombosis and systemic inflammatory response syndromes (SIRS), respectively. Pancreatitis increases the risk of deep vein thrombosis (DVT) through a combination of increased production of pro-inflammatory cytokines and systemic vascular injury. However, DVT and pulmonary embolism remain under-recognized and underappreciated complications of acute pancreatitis as they fall through the cracks in the commonly used venous thromboembolism (VTE) risk assessment model. We therefore propose that VTE prophylaxis needs to be considered by all clinicians when admitting and evaluating patients with acute pancreatitis and that acute pancreatitis needs to be included on the various VTE risk assessment calculators as it is a significant risk factor for the development of VTE.

## Introduction

Acute pancreatitis is an inflammatory condition caused by an insult to the pancreas. The most common etiologies include alcohol consumption and gallstones [1]. Pancreatitis is associated with local and systemic complications. An example of a localized complication is splenic vein thrombosis [2]; however, this is felt to be due to the close proximity of the pancreas and splenic vein, placing this particular vein at risk for damage secondary to leakage of inflammatory or proteolytic molecules. A systemic complication of acute pancreatitis is systemic inflammatory response syndrome (SIRS). This pancreatitis-induced SIRS is severe enough to cause systemic vascular injury [3]. It is this combination of endothelial damage, possible increased blood viscosity from dehydration (part of Virchow’s triad), and increased production of pro-inflammatory cytokines that can result in the development of venous thrombosis elsewhere in the body. Other pro-inflammatory gastrointestinal conditions such as ulcerative colitis and Crohn’s disease are associated with an increased risk of developing a venous thrombosis [4].

Deep vein thrombosis (DVT) and pulmonary embolism (PE) are under-recognized and underappreciated complications of acute pancreatitis. For hospitalized patients, risk assessment models for DVT, such as the Caprini Risk Score for assessing risk of DVT, are frequently used to determine individual patient requirements for prophylactic measures [5]. These venous thrombo-embolism (VTE) risk assessment models (RAM) play an important role in reducing the risk of VTE development in hospitalized patients [6]. Acute pancreatitis, however, does not feature in the risk criteria.

We report a patient with acute pancreatitis who subsequently developed deep venous thrombosis thought to be due to the combination of endothelial damage and increased inflammatory response of acute pancreatitis. We believe that, given the pro-thrombotic state induced by acute pancreatitis, and the substantial morbidity and mortality associated with VTE, this condition should be incorporated into the various risk assessment models for acute deep venous thrombosis.

## Case presentation

A 57-year-old female with a past medical history of depression and hypertension presented to the hospital with the chief complaint of intractable nausea and vomiting for three days prior to presentation in addition to chest pain that began a day prior to presentation. The chest pain was sharp, sub-sternal with radiation to the back and epigastrium. It was rated by the patient as being 10/10 in intensity. 

Vital signs on admission included a temperature of 97.8 degrees Fahrenheit, heart rate 79 beats/min, blood pressure 170/77 mmHg, and respiratory rate of 16/min with an oxygen saturation of 99% on room air. On physical exam, at the time of admission, the patient was in mild distress with epigastric tenderness noted on palpation. The remainder of the physical exam was unremarkable.

**Figure 1:**
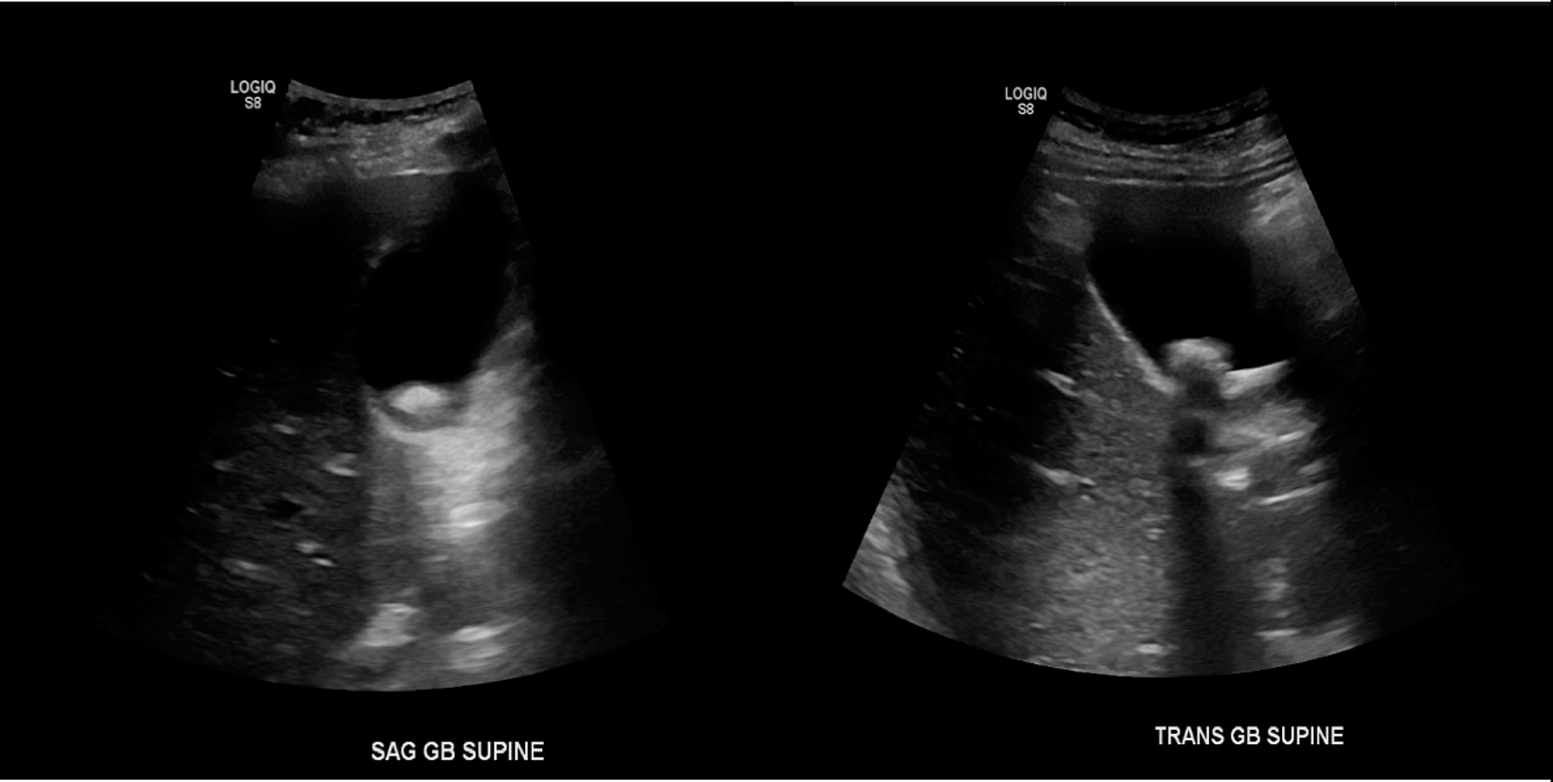
US of the abdomen demonstrating the gall bladder with no wall thickening or pericholecystic fluid. Shadowing gallstone(s) identified.

**Figure 2:**
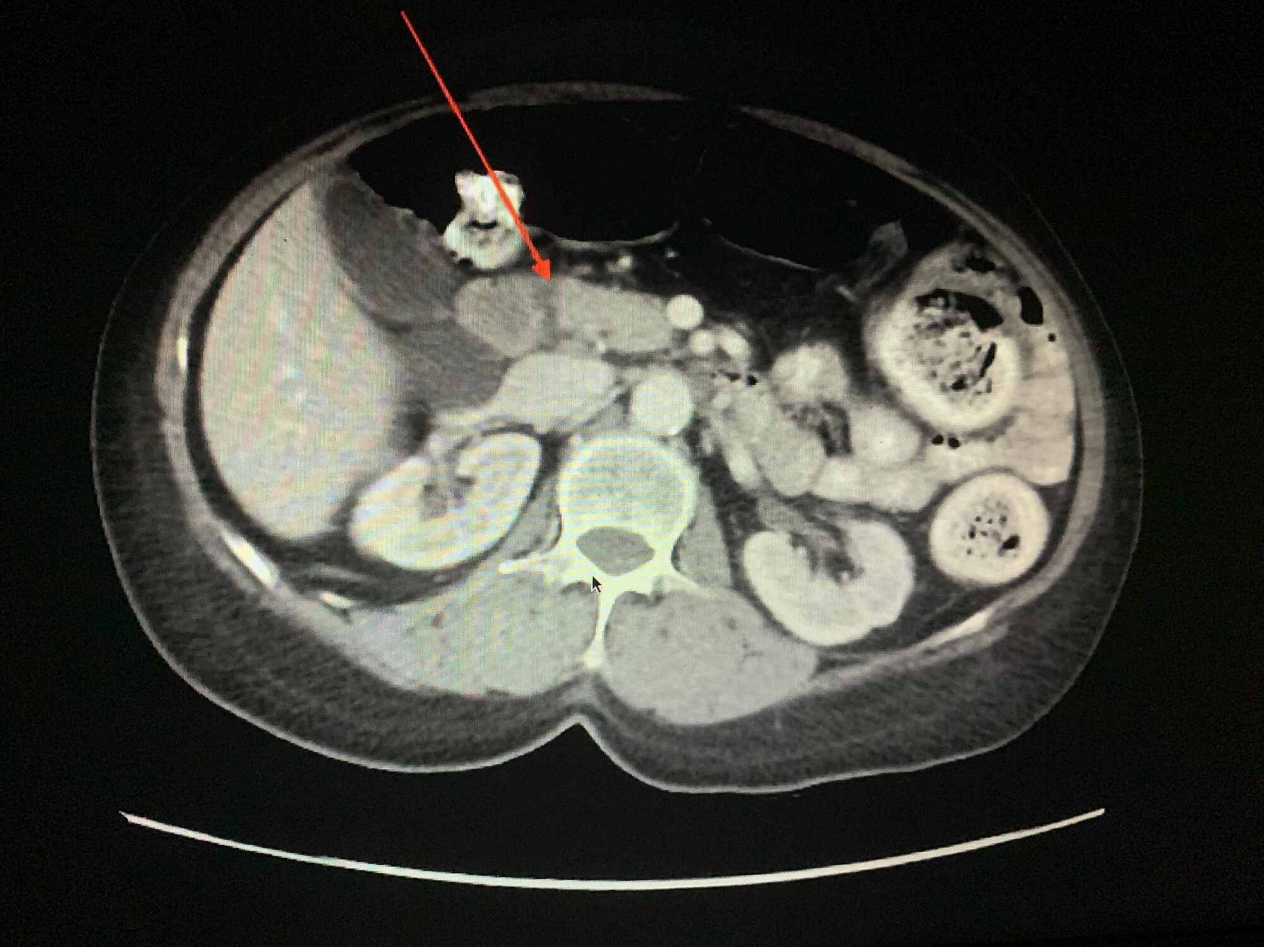
CT of the abdomen and pelvis demonstrating fat infiltration within the pancreaticoduodenal groove consistent with acute interstitial pancreatitis

Laboratory values included an elevated troponin level that peaked at 0.07ng/ml. Electrocardiogram showed normal sinus rhythm with sinus arrhythmia and non-specific ST and T wave changes. Aspartate aminotransferase (AST) and alanine aminotransferase (ALT) levels were elevated at 337 U/L and 215 U/L, respectively. Total bilirubin was normal at 0.38 mg/dL. Lipase was noted to be elevated at 1,174 U/L with triglycerides at 124 mg/dL. A right upper quadrant ultrasound showed cholelithiasis without evidence of choledocholithiasis or cholecystitis (Figure 1). Computed tomography (CT) scan of the abdomen and pelvis with contrast demonstrated fatty infiltration within the pancreaticoduodenal groove consistent with acute interstitial pancreatitis (Figure 2).

**Figure 3:**
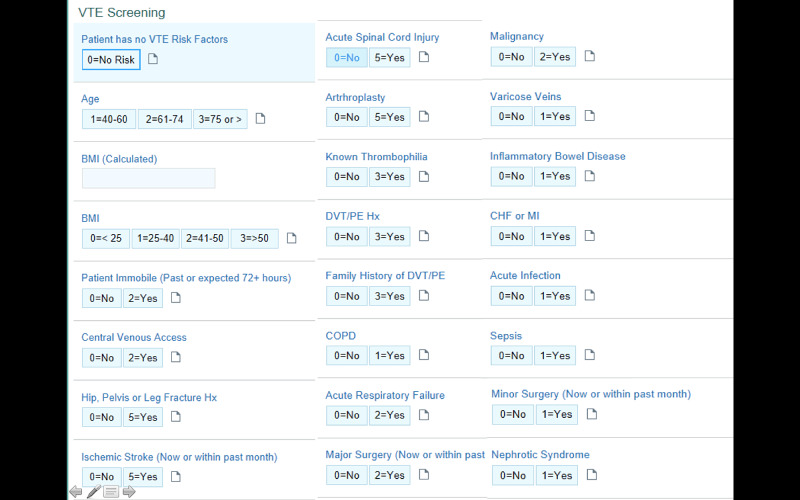
Venous thrombo-embolism (VTE) risk assessment calculator (Chemical prophylaxis indicated for score >2).

The patient improved with intravenous volume resuscitation and electrolyte replacement. Her laboratory parameters improved to lipase 503 U/L (the next day), AST 11 U/L, and ALT 52 U/L (over the course of five days). The patient was discharged after a five-day hospital stay. Further plan of care included outpatient follow-up with her primary care provider, and surgical follow-up for consideration of elective cholecystectomy. During this admission, the patient was encouraged to ambulate but was not started on chemical VTE prophylaxis due to a low score (1 for age) based on a modified version of the Caprini VTE risk assessment calculator utilized at our institution (Figure 3).

**Figure 4:**
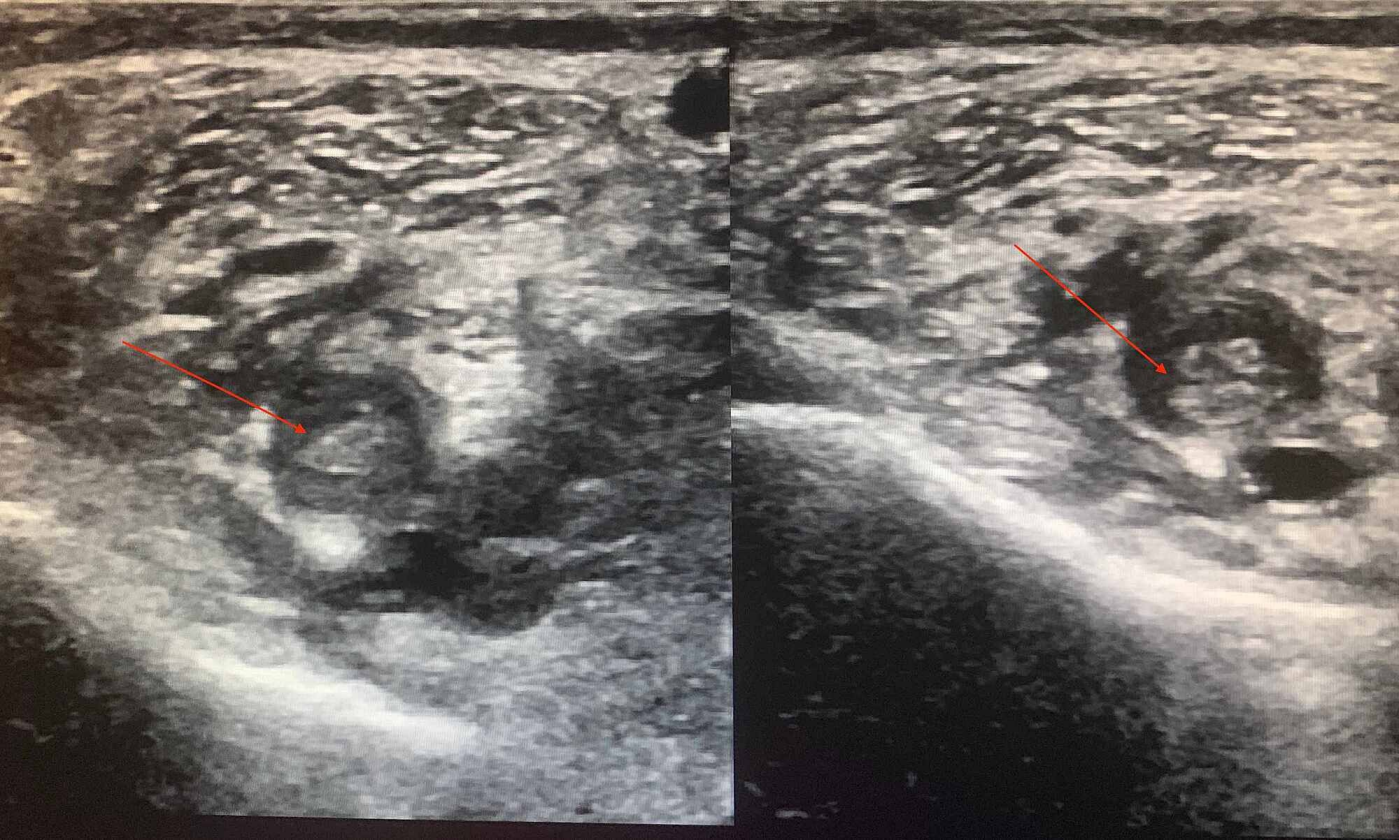
Lower Limb Venous Duplex showing deep vein thrombosis (DVT) in the popliteal vein

**Figure 5:**
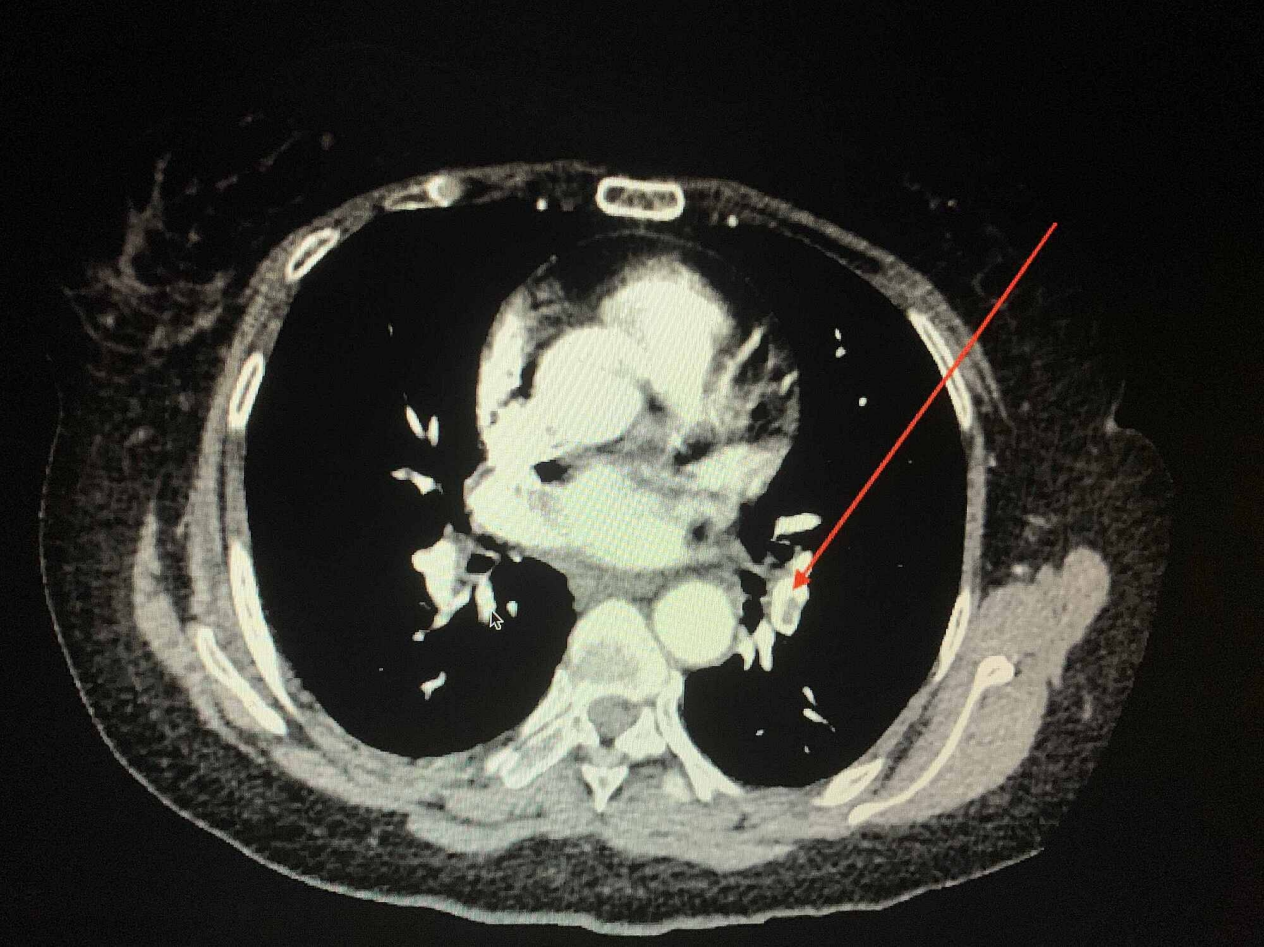
CT angiogram chest pulmonary embolism (PE) study demonstrating a small linear filling defect

She returned to the hospital three days after discharge following a visit to her primary care physician who noted a significant difference in her calf circumference with the right leg larger than the left. There was an associated Moses’ sign. Doppler ultrasound demonstrated acute occlusive deep vein thrombosis from the mid to distal femoral vein, popliteal vein and one of the posterior tibial veins in the proximal calf (Figure 4). The patient was subsequently readmitted and started on heparin infusion. While the CT angiogram showed a few small non-occlusive chronic emboli bilaterally, there was no evidence of an acute pulmonary embolus (Figure 5).

## Discussion

Validated VTE risk assessment models (RAM) such as Padua, Caprini and Geneva prediction scores [7] include co-morbidities shown to increase the risk for VTE formation such as active cancer, prior thrombotic episode, heart or respiratory conditions, infections and/or rheumatologic conditions. These RAM are effective in discriminating between medical patients at high and low risk for VTE [8]. High-risk patients should receive chemical prophylaxis, which substantially reduce their risk of developing a VTE [9]. Barbar et al. [6] using the Padua RAM, for example, showed that chemical VTE prophylaxis provided an 87% VTE risk reduction in high-risk patients who received VTE chemical prophylaxis compared to those that did not. 

Acute pancreatitis, however, falls through the cracks in these RAM as it is neither an infection, rheumatologic condition, nor is it an active malignancy. Acute pancreatitis is an inflammatory reaction. It occurs following pancreatic cell damage that results in activation and induction of pro-inflammatory cytokines, notably IL-6, IL-8, and macrophage migration inhibitory factor [10], which in turn promote further parenchymal cell damage. Inflammation is intricately involved in venous thrombus formation and inflammation is postulated to be a “stimulus” or “catalyst” for the development of a pro-thrombotic state [11]. Fox et al. systemic review of clinical studies looking at the relationship between inflammation and thrombus, and found the same inflammatory markers (IL-6 and IL-8) to be involved in the pathogenesis of VTE [12]. Umapathy et al., on the other hand, looked at 2,453,997 discharges with acute pancreatitis and found that 23,614 (1%) were associated with VTE [13-14]. The incidence of DVT and PE was almost two-fold higher in patients with acute pancreatitis than the controls irrespective of the sex, age, or comorbidity.

## Conclusions

Venous thromboembolism remains an important but under-recognized complication of acute pancreatitis. Given that 10 to 30% of people will die within one month of diagnosis of VTE and one-third to one-half will have long-term complications, preventing such events should be considered “high stakes” and the threshold for implementing preventing measures low. We therefore propose that VTE prophylaxis needs to be considered by all clinicians when admitting and evaluating patients with acute pancreatitis and that acute pancreatitis needs to be included on the various VTE risk assessment calculators as it is a significant risk factor for the development of VTE.
